# A primer of an in-depth resilience status for German medical graduates: results of a cross-sectional survey on the status quo of resilience among graduates of human medicine in Bavaria, Germany - a necessary step in building an emotionally equipped healthcare work-force

**DOI:** 10.1186/s12909-021-02933-z

**Published:** 2021-11-12

**Authors:** J. Kiesewetter, J. Huber

**Affiliations:** Institut für Didaktik und Ausbildungsforschung in der Medizin am Klinikum der LMU München, Ludwig-Maximilians-Universität München, Pettenkoferstr. 8a, 80336 Munich, Germany

**Keywords:** Resilience, Stress, Burnout, Graduates of medical studies

## Abstract

**Background:**

Resilience is a widely-used catchword in the last couple of years to describe the resistance to psychological strains of life, especially for the healthcare work-force. The promises of resilience to burnout sound great and what we all would want: less health impairment despite stress, higher work satisfaction and last but not least higher work performance. There is research that shows that students and physicians have high emotional distress and low resilience, yet comparably little is known which aspects of resilience are exactly impaired in the upcoming work-force. With our study we investigated the in-depth resilience status of medical graduates from five medical schools within their first year after graduation. In this, additionally to assessing the resilience status as a whole we investigate the answers on the singular items and the relationship of the resilience status with neighboring constructs.

**Methods:**

In 2018, 1610 human medical graduates from five Bavarian medical schools were asked to take part at cross-sectional Bavarian graduate survey (Bayerische Absolventenstudie Medizin, MediBAS). The response rate was 38,07, 60% of the participants were female. For the identification of the in-depth resilience status we included the 5-point Likert 10-Item Connor-Davidson Resilience Scale, German Version in a graduate survey posted to 5 medical schools and over 1610 eligible participants of whom 610 (60% female) filled out at least parts of the survey. To identify relationships to other aspects we posed further questionnaires.

**Results:**

The resilience status showed a mean resilience score of M = 37.1 (SD = 6.30). The score ranges from 3.22 (I am not easily discouraged by failure) to 4.26 (I am able to adapt to change). One third of the participants chose not to answer the item “I am able to handle unpleasant feeling”. Relationships to job satisfaction, scientific competence and stress are presented in the article.

**Conclusions:**

The study shows that the overall resilience status of medical graduates one year after their graduation is rather high, but subjectively they do not feel equivalently resilient for the different aspects they face in their job. Especially, how to handle their emotions seems to be challenging for some of the young physicians. In the article we sketch ideas how to handle the specific training needs the study has identified.

**Supplementary Information:**

The online version contains supplementary material available at 10.1186/s12909-021-02933-z.

## Background

Resilience is a widely-used catchword in the last couple of years to describe the resistance to psychological strains of life. The promises of resilience to burnout sound great and what we all would want: less health impairment despite stress [8], higher work satisfaction [25] and last but not least higher work performance [14]. Resilience also helps to circumvent issues such as impaired concentration, increased cynicism, undermined professional development, and jeopardized care for patients [4].

Regarding medical students and physicians it was found that higher perceived stress is related to lower resilience [1, 22]. W and that resilience negatively correlates to mental health problems [20] depressive and other stress related symptoms [9].

On the one hand, we do know that developing and maintaining of resilience is important to stay physicially and mentally healthy. On the other hand resilience has rarely been used as the goal of medical education, but has rather been implicitly trained [10]. For example a study found that higher experience of trauma in medical school is associated with personal gain [9]. The evidence thus far shows that medical students are emotionally not equipped better than their peers for their future job [22]. Surely, a more formalized and planned training of resilience for medical students and physicians is a good idea [12]. One study proposes a whole training program in order to support students [19], others see resilience more as a means to life-satisfaction [12]. We agree with the notion that the relationship to other constructs is very important to identify what we understand as resilience, but think that we need to identify even more in detail which parts of resilience are (most) in need of training. What exactly and potentially how do we want and need to influence resilience as medical educators?

With our article we would like to investigate the resilience status of graduates for human medicine from five medical schools in Bavaria in their first year after graduation.

In German and international medical education resilience in medical students has not been studied in-depth. Such an analysis is necessary to know where to shift the focus to, when training aspects of resilience in and after medical school.

Therefore, in our study, we aimed to assess medical school graduates’ resilience status in Bavaria at the first year of their working career. We investigate the overall score and the answers on singular items. Additionally, we study the relationship of resilience to other constructs. Namely, we assume that the constructs “stress,” “job satisfaction” and “scientific competence” in particular are closely linked to resilience and therefore could make it more transparent how they relate to training needs. Whether and to what extent scientific competency relates to the resilience of individuals is currently unclear. Students in Germany learn how to be a physician and medical scientist simultaneously and we assume that the acquired level of scientific competence could make it easier to study and subsequently work.

Since we want to investigate and understand the resilience status of medical graduates, it is obvious to take a closer look at stress as a much better construct and job satisfaction as a potential results of failed or successful resilience. In particular, we want to be able to provide the ground to develop appropriate measures to promote resilience. We therefore focus on the item- to- item variation of our assessment.

Our hypotheses are:

1. There is variation in resilience status and different aspects of resilience status in the cohort studied.
2. Job satisfaction, stress and scientific competence are related to the resilience status.
  - There is a positive relationship of “scientific competence” and resilience status.
  - There is a negative relationship of “stress” and resilience and a positive relationship of “job satisfaction” and resilience status.

## Methods

### Assessment of the resilience status of medical students

A survey in the course of the so called “The survey of Bavarian medical graduates (Bayerische Absolventenstudie Medizin, MediBAS)” is a multi-centric cross-sectional survey comprised of several validated questionnaires (listed below) conducted in five Bavarian medical schools (Friedrich-Alexander-Universität Erlangen-Nürnberg, Julius-Maximilians-Universität Würzburg, Ludwig-Maximilians-Universität München, Technische Universität München, Universität Regensburg) and one Bavarian veterinary medical school (Ludwig-Maximilians-Universität München). The German higher Education system is organized in a way that it ends with a state wide licensing exam for all medical professions and veterinarians. We did conduct the survey with all graduates because they were part of the same graduate pool, but did only analyze the human medicine students. Bavaria is a large state in Germany and is representative for German Medical Schools regarding the number of students enrolled and medical school sizes included. Germany has a final licensing exam after which the students are allowed to practice medicine.

The study was done from October 2018 to January 2019, roughly one year after the students final graduation. We conducted the study in collaboration of the Kompetenznetz Medizinlehre Bayern (KMB) and the Bavarian institute for higher education research and higher education planning (Bayerisches Staatsinstitut für Hochschulforschung und Hochschulplanung, IHF). Target persons were those who had finished their final state license examination between 01. October 2016 and 30. September 2017 at one of the participating faculties. The questionnaire was sent out in an online version (via Questbeck, Globalpark inc.) and a paper-based version to all participants at their home address via mail and e-mail and a the request to send it back by September 30th the latest. We pseudomized both with the same code to avoid double-participation. We will present data of the human medical graduates only. All 1610 graduates were asked to participate, 613 did participate, the response rate was 38,07, 60% of the participants were female. A very representative to the quota of female medical students of 62,5% nationwide (https://de.statista.com/statistik/daten/studie/200758/umfrage/entwicklung-der-anzahl-der-medizinstudenten), completion rate was 99,8%. Drop out could have been due to motivational issues. No incentive was given for participation.

For the identification of the resilience status we included the 10-Item Connor-Davidson Resilience Scale, German Version [24] in the survey. The 10 items measure resilience with a 5-point Likert type response scale form 0 (never) to 4 (almost always). The questionnaire represents the following aspects of resilience [3]:

- Adapt to change
- Deal with whatever comes my way
- See humorous side of things
- Stress makes me stronger
- Bounce back after illness or injury
- Believe I can achieve goals despite obstacles
- Under pressure I stay focused
- Not easily discouraged by failure
- Think of myself as a strong person when facing challenges
- Able to handle unpleasant feelings

Participants’ answers should be given based on reflection from the previous four weeks of their life. The level of agreement within each item allows for the calculation of a score for each participant. Possible overall score ranges vary from a minimum of 0 (lowest level of resilience) to a maximum of 40 (highest level of resilience). We used the German version validated by Sarubin et al. [24].

To identify how participants’ resilience level relate to other constructs we included other measures. To self assess professional competence we included the professional competence scale (13 items) of the Freiburger competence questionnaire with overall 45 items [6]. To measure the scientific competence we included the Munich Research Competence Scale [5], whereof scientific competence is based on the learning goals of the national competency based learning objective catalogue medicine (Nationaler Kompetenzbasierter Lernzielkatalog Medizin; wwww.nklm.de).

We further asked questions regarding their demography, income and working time, discrimination and mobbing experiences as well as their perception of how they deal with excessive demands, how they handle feedback and workplace learning. The latter two measures are adapted from a questionnaire for social workers [23] and have since been validated (Heim, M., Schulz, C. M., Schneider, F., Berberat, P. O., Gartmeier, M., & Schick, K., Measuring informal workplace learning outcomes in graduate medical education: validity and adaptation of an instrument suitable for residents, Submitted). All of the scales used in MediBAP and their generation is detailed in the “Methodenbericht” which can be inquired at https://www.bap.ihf.bayern.de/medibas .

All measures were very carefully chosen as part of the study to gain as much insight into the potential relationship of resilience to the other constructs. We had we had limited participant-time to allocate and therefor had to make hard choices. For example we chose to use the 10 item instead of the 25 item version of the German version of the Connor-Davidson Resilience Scale [24].

### Statistical analysis

To assess the resilience status, a mean score for each item was calculated. To further advance the knowledge on the resilience status and to detect potential confounder variables, we calculated the relationship of the scale to other measurements of the survey with Pearson correlations. Missing data was excluded by case.

In order to give a clear and complete account of our study we provide a Supplement to the study with the STROBE checklist (www.strobe-statement.org).

## Results

### Demographics

We have summarized the demographic data of our sample in Table 1.

**Table 1:** Demographic data of medical students

	Frequency	Proportion in %
Age		
20–30 years	452	73,7
30–40 years	100	16,3
> 40 years	7	1,1
Total	559	91,2
Missing	54	8,8
**Gender**		
Female	375	31,8
Male	195	61,2
Total	570	93,0
Missing	43	7,0
**Migration background**		
Yes	94	15,3
No	449	73,2
Total	543	88,6
Missing	70	11,4

### Resilience of medical students and its relationship to other constructs

1. The Connor-Davidson resilience scale resulted in a mean resilience score of M = 37.1 (SD = 0,63). As expected resilience was measured highly reliable (Cronbachs α = .88). To investigate hypothesis 1 (There is variation in resilience status and different aspects of resilience status in the cohort studied.) we analyzed the item-to-item variation in the Connor-Davidson resilience scale. We have depicted the individual scores in Table 2.

**Table 2:** Resilience scores of medical students

I am able to…	Missing	N	M	SD
Adapt to change	41	572	4.26	0.77
Deal with whatever comes my way	42	571	3.43	0.91
See humorous side of things	44	569	3.41	1.03
Stress makes me stronger	43	570	3.46	0.94
Bounce back after illness or injury	42	571	4.06	0.96
Believe I can achieve goals despite obstacles	45	568	4.14	0.85
Under pressure I stay focused	44	569	3.60	0.90
Not easily discouraged by failure	46	567	3.22	0.99
Think of myself as a strong person when facing challenges	46	567	3.78	0.91
Able to handle unpleasant feelings	195	418	3.78	0.89
Overall score sum			37.10	6.3

The score of the last item shows a high number of non-respondents. Many of the participants did choose not to answer the last item. In general the scores seem relatively high compared to other nonclinical samples [24].

To investigate hypothesis 2 (Job satisfaction, stress and scientific competence are related to the resilience status) we first correlated the overall scale to the scale of professional competence including the items of stress and job satisfaction.

Resilience status did positively relate to professional competence (13 items, correlation of r = .19; *p* < .05) and scientific competence (10 items, correlation r = .19; *p* < .05). A significant negative relation between resilience and the question how satisfied participants were with their first job (r = −.18; *p* < .01) was found. Conversely, participants’ satisfaction with their medical education did relate to their resilience scores positively (r = .12, p < .01). There was a negative significant correlation regarding participants’ stress, called subjective excessive demands in the scale (How does the following attribute apply to you: professionally overwhelmed; r = −.20; p < .01; workload r = −.10; p < .05; too many stand-by duties r = −.09; p < .05). Further, we found that participants scores’ regarding discrimination and mobbing experiences regarding their ethnicity was significantly related to resilience scores (r = .08, p < .05).

The resilience score did not relate significantly to scores of professional workplace learning (r = .08, n.s.) and feedback (r = .01, n.s.). The resilience score did not relate significantly to participants’ income (r = .04, n.s.), nor their contractual (r = .04, n.s.) or real working time (r = .05, n.s.). The results are summarized in Table 3.

**Table 3:** The results of the relationship of medical students’ resilience status. Presented are the Pearson correlations to the overall resilience scores

Scales	r	p
Professional competence	.19	<.05
Scientific competence	.19	<.05
Feedback	.01	n.s.
Professional workplace learning	.08	n.s.
Singular items	r	p
How satisfied were you with your first job?	- .18	< .01
Job satisfaction: How satisfied were you with your medical education?	.12	< .01
Stress: Excessive demand: professionally overwhelmed	−.20	< .01
Stress: Excessive demand: workload	−.10	< .05
Stress: Excessive demand: too many stand-by duties	−.09	< .05
Participants’ income	.04	n.s.
Contractual working hours	.04	n.s.
Actual working hours	.05	n.s.

## Discussion

The study is a primer on resilience after medical school with a large study population from five medical schools in Bavaria, Germany. The in-depth resilience status of medical students after their graduation and their first year in at work has been investigated and might show which aspects resilience training needs to focus on.

Our results indicate that medical graduates in their first year after graduation do estimate their resilience to be rather high. It is rather likely that their resilience does not *seem* impaired because they haven’t worked for a long period of time and their resilience has not been tested yet. Another explanation might be that more resilient graduates might have been more likely to participate. Data that contradicts this bias is that that participants’ resilience scores in itself vary. Two parts of the in-depth status are especially interesting. First, students estimate regarding discouragement by failure is rather low. Second over 150 of the participants who answered all other questions chose not to answer whether they are able to handle unpleasant feelings. Medicine does entail negative feelings for physicians and patients [17] and it is one of the core components of a resilient healthcare work-force to deal with failure [27]. As medical educators we need to address this issue within the realm of a broader resilience programs. We will now discuss the further findings before we sketch a plan how to educate how to handle discouragement by failure and handling of unpleasant feelings for those students who need it. Hypothesis 1 holds true in that there is variation in resilience status and different aspects of resilience status in the cohort studied.

The resilience status we assessed did provide some insight regarding the relationship of resilience in a broader context. As could be expected, a higher level of professional and scientific competences relates to higher levels of resilience and vice versa. Medical education needs to assess uncertainty and address uncertainty-tolerance in order to counteract these tendencies [2]. Participants who felt their stress to be more excessive did have lower resilience scores or vice versa less resilient participants felt the stress (called subjective excessive demand in the scale) to be higher. Interestingly, no relation to the actual and contractual weekly working hours was found. This provides evidence that indeed it is not the workload that is important, but the way how physicians cope with the workload [21]. Hypothesis 2a holds true in that there is a positive relationship of “scientific competence” and resilience status.

Hypothesis 2b was true in part. As there is a negative relationship of “stress” and resilience status but not a positive, but a negative relationship of “job satisfaction” and resilience status.

The negative relationship of job satisfaction and resilience can point towards the idea that resilience is built from a necessity to develop it. As there was no relationship to feedback at the workplace, workplace-based learning and the resilience score the continuing medical education put in place does not seem to suffice to advance physicians’ resilience. Resilience education might help to build resilience in undergraduate and continuing medical education [26]. But potentially generic resilience programs might not be necessary for everyone. Our data shows that especially 1) handling disencouragement by failure and 2) struggling with one’s own emotions while dealing with patients could be important key aspects that need to be learned by graduates. We will now sketch very roughly how these aspects could be learned.

1) Self-worth/self-care training: To deal with unpleasant feelings self-worth trainings incorporate practices to learn to focus, instead of avoiding, unpleasant feelings [15]. Paradoxically focusing on these feelings make them disappear. A recent study found that incorporating practices of self-care significantly reduces feelings of exhaustion and disengagement [23].

2) Normalizing emotions in medical education: The Dutch psychologist Kristin Neff proposed to embrace those parts that are most hurt [16]. For example: mindfulness can provide a tool embracing emotions without judgment and altering the cognitive and emotional experience [18, 11].

3) Emotional after-care after strong emotions: Strong emotions are the ones that come rapidly in situations out of our control. An example can be the anger a physician experiences after a patient is frustrated with the physician because they disagree on the treatment plan. Physicians should be educated to seek external help to deal with these strong emotions – in form of Balint-group intervention, supervision or therapy. In psychiatry or psychosomatics this has been standard practice for a long time. Yet, to counteract burnout in the healthcare workforce, it is of major importance to teach that in order to be medical professionals physicians who strictly work somatically also need emotional after-care (Meier, Back & Morrison, 2001).

For all of these training aspects, more research is needed to see whether the porposed plan does indeed infliuence the items we see in focus in medical education. As for now research on resilience training for medical students has oftentimes fallen short of making the methology or data completely transparent [26, 19] or has been making it transparent but replaced it with another construct like mindfulness [7]. Instead of relating resilience with other constructs we know now what aspects of resilience are missing in medical students. Now it is time to find methods to medical students training how to handle negative feelings and their discouragement by failure and investigate it with randomized controlled trials.

Not only in medical education, but also in other fields of higher education there is a growing need to embed resilience trainings. Medical education could take the lead in resilience training through setting an example of courageously addressing a major inner-professional burnout crisis.

### Limitations

Regarding the resilience status of medical graduates, we utilized a sample from graduates from Bavarian medical schools and we can’t guarantee for their representativeness, neither for medical students nor for medical graduates worldwide. Despite the low response rate of 38,07% it is a with over 600 participants it is the largest sample for Germany and one of the largest samples for assessing in-depth resilience after medical school worldwide that we know of. We provided participants with an option to fill out a paper and an online version of the questionnaire and as our study was part of the MediBAP Panel the original data was handled via a data trustee and as part of the pseudonomization the mode of conduct was eliminated. Therefor unfortunately we can’t compare the two modes of conduct and can’t rule out potential bias.

We have provided correlations for resilience with various other measurements from the survey and of course correlation does not answer questions regarding causation. Also, an interesting problem arose during the analysis of the resilience scale, which we would like to draw attention to here. There was a conspicuous non-response to the item “Able to handle unpleasant feelings” within the resilience scale. 195 respondents did not answer this question. All other questions in the scale showed a low number of missing values. The reasons for this non-response can be manifold and cannot be determined. It also remains unclear whether this was a structured non-response to the question. Therefore, a method for the imputation of the missing values was omitted. Nonetheless, we are only beginning to understand resilience in medical school and the resilience status presented here has provided us with an idea where to look at in further research. More research is needed in order to investigate which coping mechanisms graduates and medical students have in order to create evidence regarding the external validity of their estimate. Methodologically, the 10 item CD-RISC is a good first step and it was useful to look deeper into the data. Further investigations should use the 25 item Version of the CD-RISC scale. Furthermore, a longitudinal study could have provided more insight into the development of resilience.

## Conclusions

For medicine, by gaining increased insight into resilience status, we need to take the advancement of individualized emotional coping mechanisms of medical students and healthcare professionals seriously [10]. With one-year graduates having medium to high resilience scores, but struggling how to deal with one’s own emotions medical education should make this the focal point of scientific discovery and training to counteract burnout tendencies in the medical profession.

## Supplementary Information

**Additional file 1.** STROBE Statement—checklist of items that should be included in reports of observational studies.

## Data Availability

The MediBAS data can be requested from the Bavarian Institute of Higher Education Research and Planning (www.ihf.bayern.de).

## Abbreviations

MediBAS: Bayerische Absolventenstudie Medizin
KMB: Kompetenznetz Medizinlehre Bayern
IHF: Bavarian institute for higher education research and higher education planning
